# Editorial Correspondence

**Published:** 1877-09

**Authors:** F. L. Merriwethe

**Affiliations:** Daly, Texas


### EDITORIAL CORRESPONDENCE.

DALY, TEXAS.

*Editor Atlanta Medical and Surgical Journal:*

DEAR SIR: I herewith send you a report of the excision of a large fatty tumor, based on the parotid gland, and occupying the whole region round about the angle of the inferior maxilliary bone.

I don't send it for any very peculiar interesting features connected with the tumor, or the operation for its removal, but to add another to the reported operations in which chloroform was used as an anæsthetic and carbolic acid as antiseptic for the dressing. It is true that the connection with arteries of considerable size rendered the operation somewhat difficult, not to say dangerous. The temporal artery and blood-vessels affording nourishment to the adventitious growth —the latter being much enlarged by this service—necessarily gave some embarrassment in the operation.

Antiseptic operations attract some attention of late, perhaps justly so, and it is only by experience that useful facts are well established. Simple cases, therefore, become important to the profession. Anæsthesia by chloroform is, by some surgeons, used with hesitation, owing to unpleasant and even fatal effects in their hands. The danger probably, consists principally in a want of due caution in its use, and if accurate reports were more frequently made, an entirely safe plan might be settled upon.

The patient, a colored woman, about fifty years old, was brought thoroughly under the influence of chloroform, when, assisted by Drs. Smith, Crocket, Dupre, and L. Merriwether, I commenced the operation by making an elliptical incision from the angle of the jaw to the mastoid process. Notwithstanding the care with which the tumor was dissected out from its bed, the temporal and other smaller arteries required ligation. Very little blood was lost, and after bringing the edges of the incision together with sutures, the carbolic dressing was applied, with the effect of preventing undue inflammation, and securing ready union.

Thus the old woman was relived, without suffering or other unpleasant consequences, of a troublesome tumor which had already made impression upon her constitution.

F. L. MERRIWETHE, M.D.

